# Intracellular Clusterin Interacts with Brain Isoforms of the Bridging Integrator 1 and with the Microtubule-Associated Protein Tau in Alzheimer's Disease

**DOI:** 10.1371/journal.pone.0103187

**Published:** 2014-07-22

**Authors:** Yuan Zhou, Ikuo Hayashi, Jacky Wong, Katherine Tugusheva, John J. Renger, Celina Zerbinatti

**Affiliations:** Department of Neuroscience, Early Development and Discovery Sciences, Merck Research Laboratories, Merck Sharp & Dohme Corp., West Point, Pennsylvania, United States of America; The University of Queensland, Australia

## Abstract

Sporadic or late-onset Alzheimer's disease (AD) is expected to affect 50% of individuals reaching 85 years of age. The most significant genetic risk factor for late-onset AD is the *e4* allele of *APOE* gene encoding apolipoprotein E, a lipid carrier shown to modulate brain amyloid burden. Recent genome-wide association studies have uncovered additional single nucleotide polymorphisms (SNPs) linked to AD susceptibility, including those in the *CLU* and *BIN1* genes encoding for clusterin (CLU) and the bridging integrator 1 (BIN1) proteins, respectively. Because CLU has been implicated in brain amyloid-β (Aβ) clearance in mouse models of amyloid deposition, we sought to investigate whether an AD-linked SNP in the *CLU* gene altered Aβ42 biomarker levels in the cerebrospinal fluid (CSF). Instead, we found that the *CLU rs11136000* SNP modified CSF levels of the microtubule-associated protein Tau in AD patients. We also found that an intracellular form of CLU (iCLU) was upregulated in the brain of Tau overexpressing Tg4510 mice, but not in Tg2576 amyloid mouse model. By overexpressing iCLU and Tau in cell culture systems we discovered that iCLU was a Tau-interacting protein and that iCLU associated with brain-specific isoforms of BIN1, also recently identified as a Tau-binding protein. Through expression analysis of CLU and BIN1 variants, we found that CLU and BIN1 interacted via their coiled-coil motifs. In co-immunoprecipitation studies using human brain tissue, we showed that iCLU and the major BIN1 isoform expressed in neurons were associated with modified Tau species found in AD. Finally, we showed that expression of certain coding *CLU* variants linked to AD risk led to increased levels of iCLU. Together, our findings suggest that iCLU and BIN1 interaction might impact Tau function in neurons and uncover potential new mechanisms underlying the etiology of Tau pathology in AD.

## Introduction

Multiple genome-wide association studies (GWAS) have replicated a link between common single nucleotide polymorphisms in the *CLU* gene (*rs11136000* and *rs1532278*) and increased susceptibility for late-onset Alzheimer's disease (AD) [1]–[7]. In addition, rare *CLU* variants revealed by next-generation sequencing have also been associated with AD risk [8]. However, the mechanisms by which modifications in clusterin expression and/or function alter disease risk are not yet clear [9]. Clusterin (CLU) is synthesized as a 60–80 kD precursor protein that undergoes internal cleavage generating α- and β-chains joined by disulfide bonds [10]. This glycosylated heterodimeric CLU is constitutively secreted and referred to as soluble clusterin (sCLU), or as apolipoprotein J (apoJ), when found in association with lipoproteins [11]. Shorter forms of the precursor CLU have been detected intracellularly and named cytosolic, truncated or nuclear CLU [12]–[14]. Alternative splicing, internal translation initiation, mistranslocation of sCLU, and impaired proteasomal degradation all appear to contribute to the pool of cytosolic CLU isoforms [14]. The function of intracellular CLU (iCLU) is not completely understood. Studies in cancer biology have linked iCLU to Bax-mediated apoptosis [15], [16]. Of relevance to AD, it has been recently shown that iCLU levels increase quickly in cultured primary neurons exposed to amyloid-β peptides (Aβ), and that this iCLU elevation is required for the neurotoxic downstream signaling effects of Aβ [17].

CLU expression is highest in the brain and is markedly upregulated under situations of stress and inflammation [18], [19]. Induction of CLU mRNA is observed within pyramidal neurons of the hippocampus and the entorhinal cortex of AD patients [20], [21], and CLU immunoreactivity is found in association with neutrophil threads, neurofibrillary tangles and amyloid plaques [20]. Published literature suggests that CLU plays a chaperone role for Aβ, modulating both its clearance and deposition [21]–[23], similar to a function proposed for apoE [24]. Seminal support for this putative role of CLU was revealed by the double deletion of CLU and apoE in a mouse model of amyloid deposition [23]. While no substantial changes in brain amyloid were detected with the individual deletions, double knockout (KO) mice showed a dramatic exacerbation of amyloid burden. The hypothesis is further substantiated by findings showing direct interaction between CLU and Aβ [25], [26]. However, despite these suggestive preclinical findings, clinical data is lacking to corroborate a significant effect of CLU on Aβ burden as a major mechanism underlying the genetic link to AD [27], [28]. Healthy carriers of the *CLU rs11136000* risk allele *C* show decreased white matter integrity [29], altered coupling between hippocampus and prefrontal cortex during memory processing [30], and significant longitudinal increases of cerebral blood flow in the hippocampus and anterior cingulate cortex [31], indicating that CLU may also participate in non-Aβ pathways that could modulate vulnerability to AD.

First identified as a tumor suppressor [32], the bridging integrator 1 (BIN1) has been recently linked to AD susceptibility by GWAS [33]–[37]. BIN1 is highly expressed in the brain and all seven brain-specific BIN1 isoforms have an inserted domain that interacts with clathrin and AP2/α-adaptin (CLAP), indicating a key role for neuronal BIN1 in endocytosis [38]. The exact mechanism by which polymorphisms in the *BIN1* gene alter AD risk is still unknown, but a recent study provided evidence that BIN1 interacts with the microtubule-associated protein Tau [39]. BIN1 immunoreactivity was found in co-localization with neurofibrillary tangles in the AD brain, and knockdown of the *BIN1* ortholog *Amph* partially restored the rough eye phenotype associated with human Tau overexpression in *Drosophila* [39].

Here we provide evidence that the *CLU rs11136000* SNP alters CSF Tau levels in AD, and that iCLU is a Tau-interacting protein elevated in the brain of Tau-overexpressing Tg4510 mice. We also demonstrate that iCLU interacts with brain-specific BIN1 isoforms containing a putative coiled-coil motif. Furthermore, an AD-risk iCLU mutant lacking the *C*-terminus coiled-coil motif does not interact with BIN1, suggesting that CLU and BIN1 association is mediated via their coiled-coil domains. Lastly, we show that iCLU and BIN1 isoform 1 are associated with modified Tau species found in the brain of AD patients, and that certain coding *CLU* mutations linked to AD risk increase the ratio between iCLU and sCLU when transfected in cells. Together, these genetic and functional studies establish a novel link between two major AD-susceptibility genes, and suggest that dysfunctions in these new Tau-interacting proteins could contribute to the etiology of AD.

## Results

### *CLU rs11136000* alters CSF Tau in AD patients

**Figure 1:**
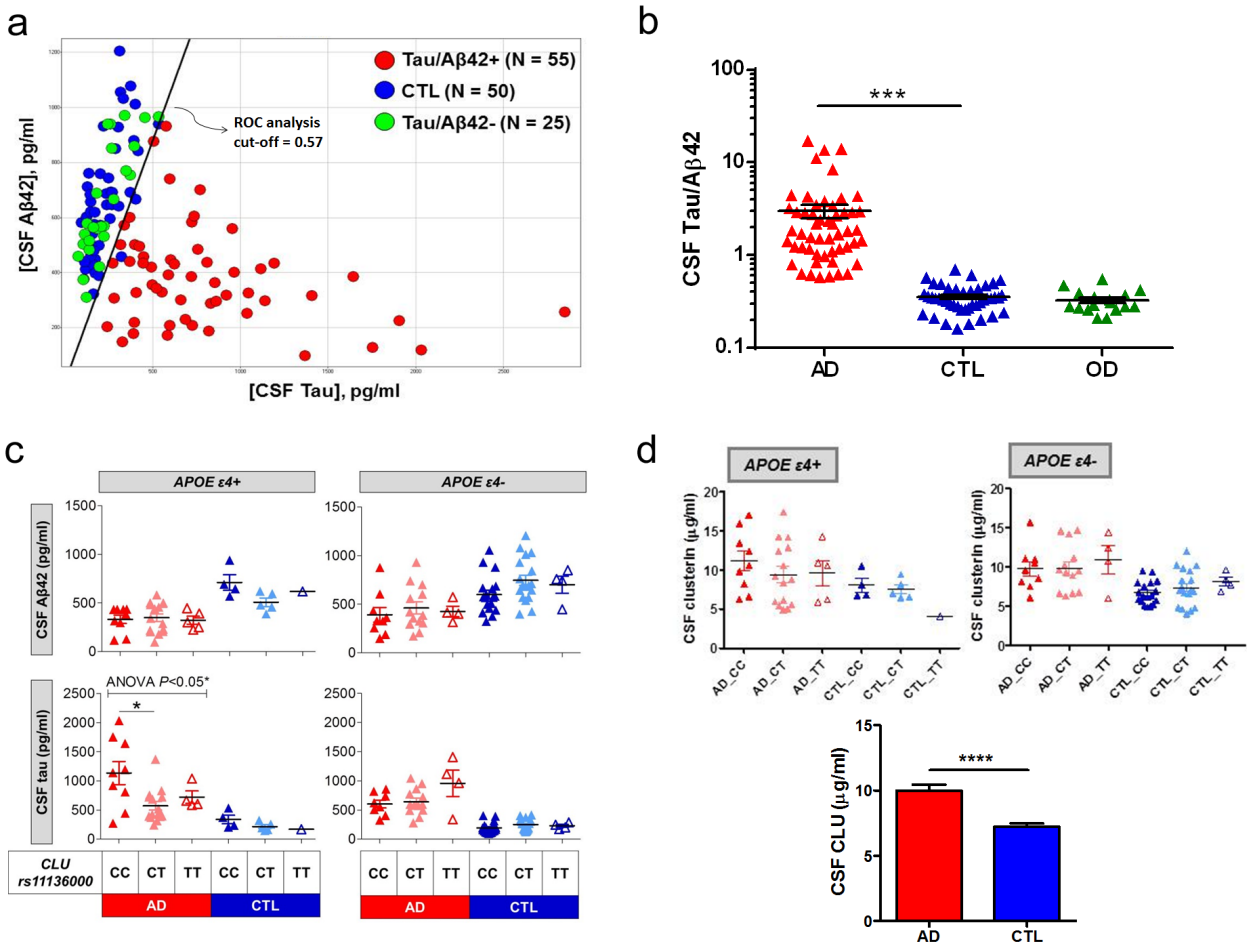
The *CLU rs11136000* risk allele *C* is associated with elevated CSF Tau in AD patients carrying apoE4. (a) Probable AD patients were divided into CSF Tau/Aβ42-positive and -negative populations using CSF Tau/Aβ42 ratio cut-off determined by ROC analysis. (b) CSF biomarker profile of control, AD and other dementia (OD) groups. (c) While no changes in CSF Aβ42 were observed as a function of the *rs11136000 CLU* polymorphism, AD patients who were apoE4 carriers and homozygous for the *CLU* risk allele *C* had significantly increased CSF Tau levels when compared to *CT* carriers. (d) No effects of CLU genotypes were observed on CSF CLU levels (top), but overall CLU protein was significantly increased in the CSF of AD compared to control subjects (bottom). *P<0.05; ***P<0.001; ****P<0.0001, Mann-Whitney test.

To assess potential mechanisms by which CLU confers risk for AD, we examined effects of the *CLU rs11136000* SNP on CSF Aβ42 and Tau levels, which are reliable disease biomarkers recently used as endophenotypes in AD genetic studies [40]. For this analysis, the AD population was enriched with patients presenting a disease CSF Tau/Aβ42 profile, which has a high diagnostic accuracy for AD over other types of dementia [41], [42]. The CSF from an initial set of 80 patients diagnosed with probable AD and 50 control non-demented individuals was evaluated for Aβ42 and Tau content by specific ELISAs. Using receiving operating curve (ROC) analysis of CSF Tau/Aβ42 ratio to establish a cut-off value (Fig. 1a), we identified 25 clinically diagnosed demented individuals who lacked the projected CSF AD profile, and were therefore subtracted from experimental patient population (Fig. 1b). Because of previously reported effects of apoE genotype on brain amyloid burden [24], we also characterized subjects for their *APOE* variants. When stratified by *rs11136000* and *APOE* genotypes, we found a significant effect of the risk allele *C* on CSF Tau levels in AD patients carrying apoE4, with homozygous *CC* individuals showing significantly higher CSF Tau than heterozygous *CT* carriers (Fig. 1c). Consistent with previous reports [43], [44], no significant *CLU* genotype effects were observed on CSF Aβ42 levels within AD patients. In addition, CSF AD biomarkers Aβ42 and Tau were not affected by the CLU genotypes in either apoE4 or non-apoE4 control subjects (Fig. 1c). While CSF CLU protein levels were not impacted by *CLU* genotypes, Tau/Aβ42-confirmed AD patients had significantly higher CSF CLU than non-demented controls (Fig. 1d), consistent with previous literature findings [45], [46]. Based on these initial results, we hypothesized a potential link between CLU and Tau pathology in AD.

### CLU is upregulated in the brain of Tg4510 Tau mouse model

**Figure 2:**
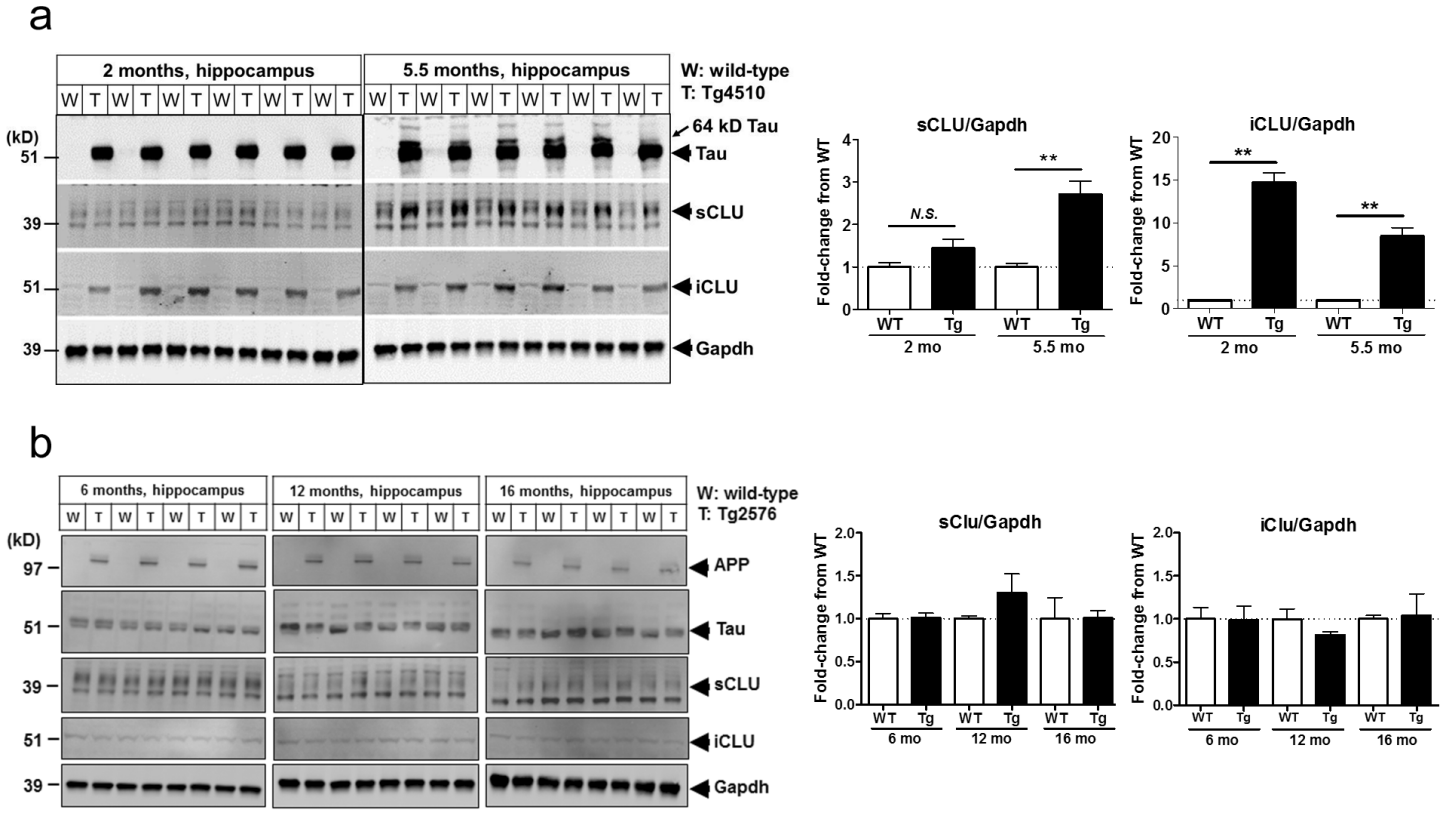
iCLU and sCLU are upregulated in the brain of a mutant Tau-overexpressing mouse model. (a) Western blotting analysis of hippocampal lysates showed upregulation of the protective sCLU in 5.5 month-old Tg4510 mice displaying overt neurodegeneration, while iCLU was markedly increased in both 2 month-old pre-tangle and 5.5 month-old Tg4510 mice. (b) No changes in sCLU or iCLU were detected by Western blotting analysis of hippocampus from Tg2576 amyloid mouse model at 6, 12 or 16 months of age. Semi-quantitative analyses of blots are shown on the right. **P<0.01, Mann-Whitney test.

We next evaluated the expression pattern of CLU isoforms in the hippocampus and cortex of Tg4510 mice overexpressing the human mutant P301L Tau associated with frontotemporal dementia [47]. While 5.5 month-old Tg4510 mice displaying Tau pathology and neurodegeneration showed marked upregulation of sCLU protein (∼30–40 kD monomer) in the hippocampus, we found that a truncated iCLU form (45–50 kD) was significantly elevated in the hippocampus of both 2 month-old pre-tangle and 5.5 month-old tangle-bearing Tg4510 in comparison to age-matched wild type (WT) mice (Fig. 2a). Unlike the clear changes in brain CLU observed with Tau overexpression, Western blot evaluation of hippocampus from Tg2576 amyloid mouse model showed no age-dependent changes in either forms of CLU compared to WT littermate controls (Fig. 2b). These findings further substantiated our hypothesis for a potential physiologically relevant connection between iCLU and Tau.

### iCLU is localized to the cytoskeleton fraction and interacts with Tau

**Figure 3:**
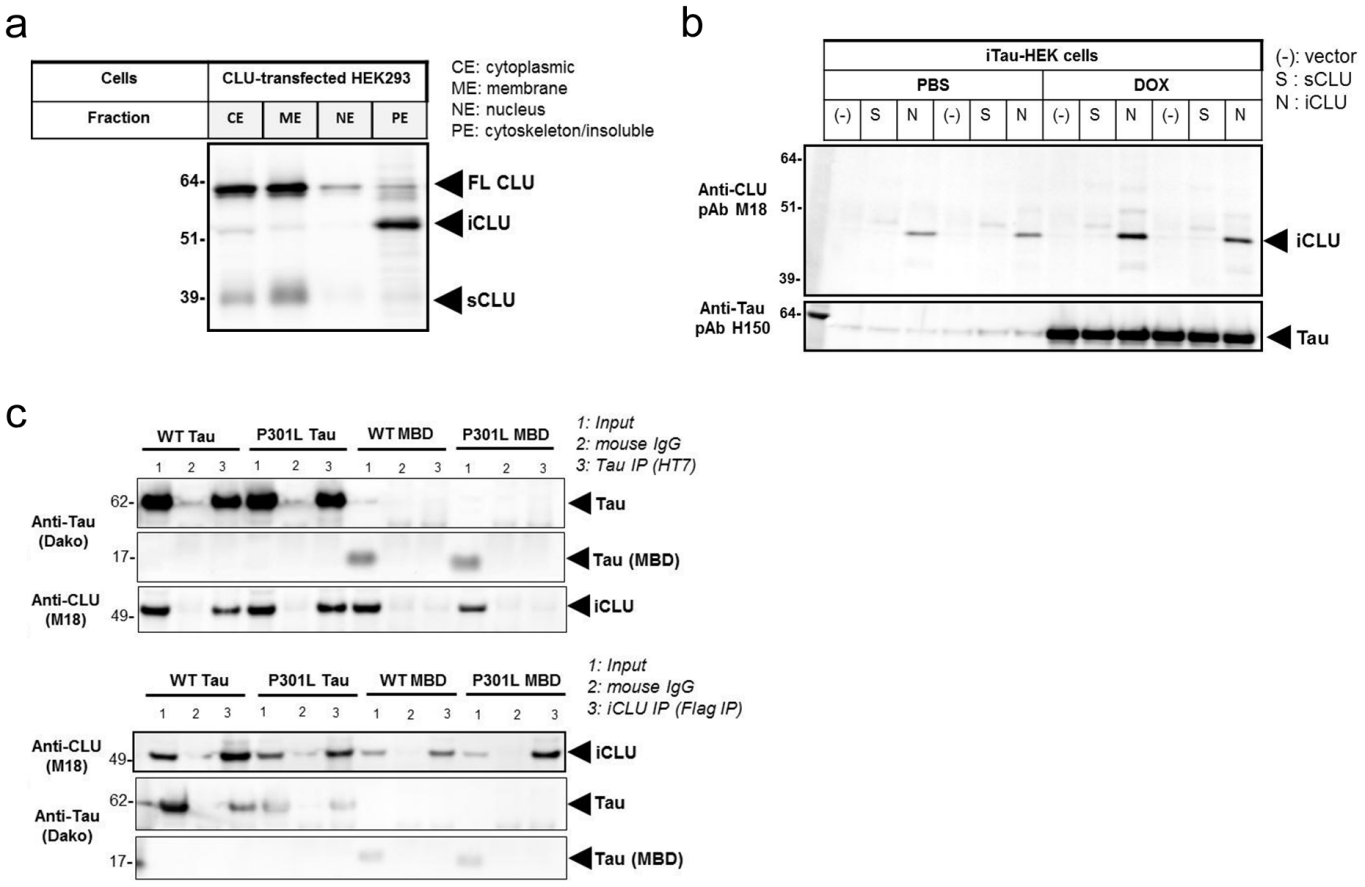
iCLU localizes to the cytoskeleton fraction and is a Tau-interacting protein. (a) Subcellular fractionation of HEK 293T after transfection with full-length CLU cDNA indicated that most of iCLU was found in the cytoskeleton/insoluble fraction. (b) Transfection of sCLU or iCLU cDNA in Tau-inducible HEK293 cells (iTau-HEK) followed by pull-down with anti-Tau antibody (HT7) showed co-immunoprecipitation of iCLU. (c) Transient co-transfection of Flag-tagged iCLU construct with WT or P301L mutant full-length Tau in HEK 293T cells followed by pull-down with anti-Tau antibody (HT7) or anti-Flag antibody showed reciprocal co-immunoprecipitation of iCLU and Tau, respectively. Conversely, transient co-transfection of Flag-tagged iCLU with WT or P301L 4-repeat microtubule binding domain (MBD) of Tau alone did not result in co-immunoprecipitation of Tau by anti-Flag antibody.

Because iCLU had been previously detected in both cytosol and nucleus, we first examined the subcellular fractionation of CLU following transfection of full-length or truncated CLU cDNA constructs in HEK 293T cells (Fig. 3a). Interestingly, we found that most of the ∼52 kD iCLU was detected in the cytoskeleton/insoluble fraction. The precursor CLU protein (∼64 kD) was fractioned primarily to the cytosol, as predicted by ER/Golgi localization. While the majority of sCLU (∼37 kD) was localized to the membrane fraction, likely associated with secretory Golgi vesicles, some sCLU was also detected in the cytosolic fraction. The primary localization of overexpressed iCLU to the cytoskeleton fraction led us to postulate the possibility of a direct interaction between iCLU and the microtubule-associated protein Tau.

To further investigate a potential interaction between CLU and Tau, we used a doxycycline (dox)-inducible wild-type Tau HEK 293T (iTau-HEK) cell line transfected with either full-length or truncated CLU cDNA constructs. By overexpressing the truncated CLU construct designed to generate only the iCLU 50 kD isoform followed by pull-down with anti-Tau antibody, we were able to co-precipitate iCLU from iTau-HEK cell lysates (Fig. 3b). Induction of Tau expression with dox increased Tau interaction with iCLU (Fig. 3b). In reciprocal co-immunoprecipitation experiments, we also evaluated whether Tau harboring the P301L mutation linked to frontotemporal dementia and overexpressed in Tg4510 mouse brain could interact with iCLU. Co-transfection of Flag-tagged iCLU and WT or P301L mutant 4-repeat full-length (4R2N) Tau into HEK 293T cells followed by pull-down with anti-Tau antibody revealed that both WT and mutant P301L Tau interacted similarly with iCLU (Fig. 3c). Reversely, pull-down of iCLU with anti-Flag antibody co-immunoprecipitated both WT and P301L full-length Tau, but not truncated Tau forms containing only the 4-repeat microtubule binding domain (MBD) (Fig. 3c). Together, these cellular studies indicated that iCLU localizes primarily to the cytoskeleton fraction of cellular extracts and interacts with both WT and P301L mutant full-length Tau. The iCLU-Tau interaction was detected in both Tau-inducible stable cell lines and in transient Tau transfection experiments, and was confirmed by reciprocal co-immunoprecipitation. In addition, our results also showed that the 4-repeat MBD of Tau alone did not show interaction with iCLU.

### iCLU interacts with brain-specific isoforms of BIN1 containing a coiled-coil motif

**Figure 4:**
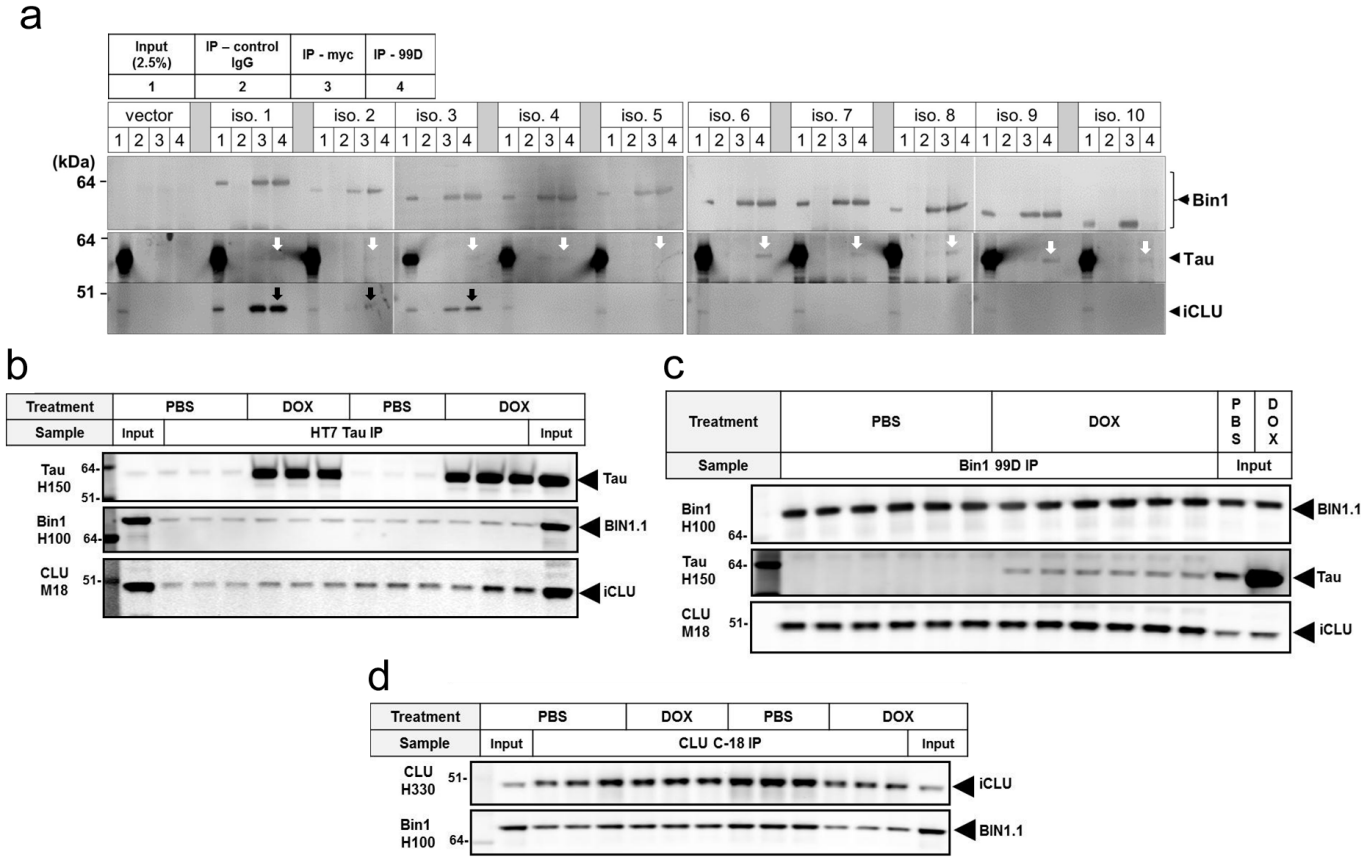
iCLU interacts with coiled-coil-containing BIN1 isoforms. (a) Co-expression of each of the ten BIN1 human isoforms with iCLU in iTau-HEK cells revealed that iCLU interacted only with brain-specific BIN1 isoforms 1–3 containing a putative coiled-coil motif within the BAR domain (black arrows); however, all isoforms of BIN1 showed interaction with Tau (white arrows). (b) Western blotting of lysates immunoprecipitated with anti-Tau HT7 antibody indicated that both iCLU and BIN1.1 were co-immunoprecipitated from both PBS and dox-treated cells. (c) Western blotting from BIN1 99 antibody pull-down showed co-precipitation of iCLU in both PBS and dox conditions, but Tau bands were only detected in dox-treated cells. (d) Western blotting of anti-CLU C18 antibody immunoprecipitates showed similar bands for BIN1.1 in PBS and dox-treated cells.

It has been recently shown that BIN1, a BAR-protein highly expressed in the brain and recently linked to AD risk, is a Tau-interacting protein [39]. BIN1 was found to co-immunoprecipitate with Tau following overexpression of both proteins in SY5Y neuroblastoma cells, and an interaction between endogenous BIN1 and Tau was also demonstrated to occur in synaptosomes from mouse brain [39]. Ten isoforms of human BIN1 are produced by alternative splicing of the *BIN1* gene [38]. Isoforms 1–7 are brain-specific and contain a CLAP domain involved in endocytosis. In addition, brain isoforms 1–3 contain a 31 amino acid insert within the BAR domain coding a putative coiled-coil region [38]. CLU has 2 coiled-coil motifs and has been reported to interact with other coiled-coil containing proteins, including the apoptosis-related Ku70 [48] and the microtubule-destabilizing neuronal protein SCLIP (SCG10-like protein) [49]. In light of this evidence, we sought to investigate whether iCLU interacted with coiled-coil containing BIN1 isoforms, and if BIN1 association with Tau was altered upon overexpression of iCLU. Following co-transfection of iCLU with each one of the ten myc-tagged BIN1 isoforms in iTau-HEK cells, we found that iCLU interacted exclusively with BIN1 isoforms 1–3, suggesting that association between iCLU and specific BIN1 isoforms depended on the presence of a coiled-coil motif (Fig. 4a). Despite the selective interaction between iCLU and BIN isoforms 1–3, all 10 isoforms of BIN1 co-immunoprecipitated with Tau. These results suggested that BIN1 interaction with Tau is not mediated by the coil-coiled motif or the CLAP domain of BIN1, and it is independent of interaction with iCLU. Next we further examined iCLU, BIN1 isoform 1 (BIN1.1) and Tau interactions in iTau-HEK cells. Upon co-transfection of iCLU and BIN1.1 in iTau-HEK cells, association of all three proteins was observed when lysates were immunoprecipitated with anti-Tau antibody, either upon overexpression of Tau (DOX) or with endogenous levels of Tau (PBS) (Fig. 4b). Independent of dox treatment, pull-down of cell lysates with anti-BIN1 antibody led to co-immunoprecipitation of iCLU, but co-immunoprecipitated Tau was detected only in dox-treated cells (Fig. 4c). When anti-CLU antibody was used for the immunoprecipitation step, BIN1.1 was pulled-down independently of dox treatment (Fig. 4d). Overall, these results suggested that the interaction between iCLU and BIN1.1 appears to occur independently of their association with Tau. In summary, our cellular findings suggested that two important genetic susceptibility factors for AD, BIN1 and CLU, are interacting proteins that also bind to the microtubule-associated protein Tau.

### CLU interacts with BIN1.1 and with modified Tau species in AD brains

**Figure 5:**
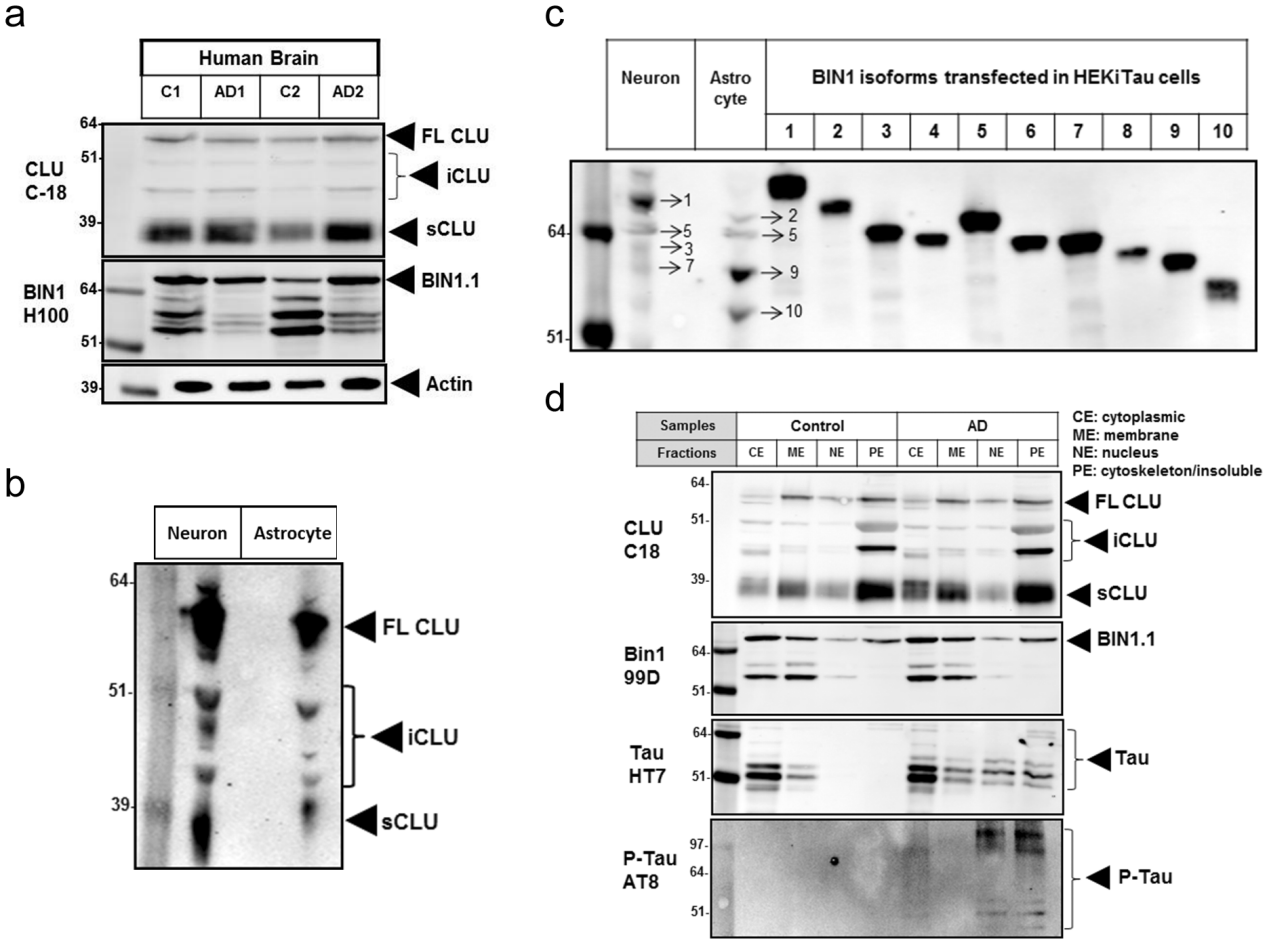
Expression and subcellular localization of CLU and BIN1 isoforms in the human cortex. (a) Western blotting detection of CLU and BIN1 isoforms found in human brain lysates. (b) Western blotting analysis of human neuronal and astrocyte primary cultures revealed significant detection of iCLU forms in both cell types. (c) Cultured human neurons express primarily brain-specific BIN1 isoform 1, and appear to lack the shorter ubiquitous BIN1 isoforms 9 and 10, which are primarily represented in astrocytes. (d) Subcellular fractionation of human brain lysates showing that BIN1.1 and CLU co-localize with Tau and p-Tau in the cytoskeleton/insoluble fraction from AD, but not control brains.

The interaction between CLU, BIN1 and Tau was further examined in brain tissue from control and AD patients. Direct Western blotting analysis showed that, in addition to the precursor and sCLU, iCLU isoforms were also detected in the human brain (Fig. 5a). BIN1 isoforms 1, 2, 5, 7, 9 and 10 were detected in whole brain homogenates (Fig. 5a). Further analysis of human neuron and astrocyte primary cell cultures revealed that iCLU forms are detected in both brain cell types (Fig. 5b). In addition, the longer brain-specific BIN1 isoforms 1–5 were predominantly detected in neurons, while the shorter ubiquitous isoforms 9 and 10 were mainly found in astrocytes (Fig. 5c). Subcellular fractionation of human brain tissue indicated that intracellular forms of CLU were preferentially associated with the cytoskeleton/insoluble fraction from brain extracts of both AD and control subjects (Fig. 5d). While most of BIN1.1 was detected in the cytosolic and membrane fractions, a significant amount of BIN1.1 was found in the cytoskeleton/insoluble fraction from control and diseased human brain (Fig. 5d). In AD brains, but not in controls, Tau and p-Tau species were also localized to the cytoskeleton/insoluble fraction (Fig. 5d).

**Figure 6:**
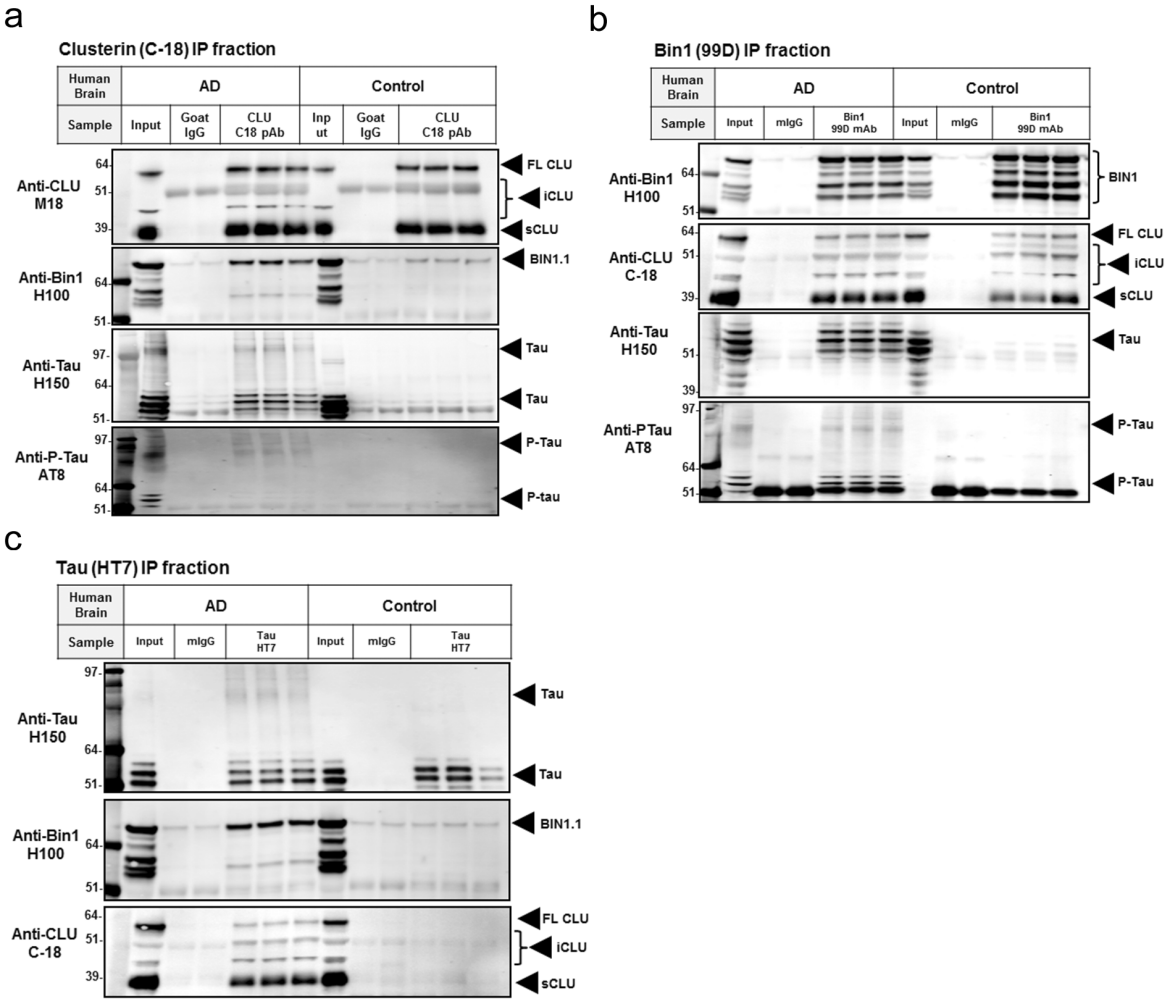
Increased interaction between CLU, BIN1 and Tau in lysates from human AD brain. (a) Immunoprecipitation of human brain lysates with anti-CLU antibody showed increased pull-down of BIN1.1 and Tau in AD compared to controls. (b) More sCLU, Tau and p-Tau were immunoprecipitated with anti-BIN1 antibody from AD than from control brain lysates. (c) Immunoprecipitation of brain lysates with anti-Tau antibody led to greater pull-down of BIN1.1 and CLU from AD than from control subjects. Results are representative of 3 AD and 3 control brains analyzed.

Since iCLU, BIN1, and Tau were all co-localized to the cytoskeleton/insoluble fraction in the AD brain, we sought to confirm our cellular findings using the co-immunoprecipitation approach with human brain lysates. While input fractions from both disease (n = 3) and control brains (n = 3) were comparable for all proteins analyzed, immunoprecipitation of brain lysates with anti-CLU antibody showed increased pull-down of BIN1.1 and Tau in AD compared to controls (Fig. 6a). Similarly, more CLU, Tau and p-Tau were immunoprecipitated from AD than from control brain lysates with anti-BIN1 antibody (Fig. 6b). Finally, immunoprecipitation of lysates with anti-Tau antibody led to greater pull-down of BIN1.1 and CLU from AD than from control tissue (Fig. 6c). Together, these findings support a relevant interaction between CLU, BIN1.1 and Tau in the AD brain.

### Rare coding CLU variants associated with AD alter sCLU/iCLU ratio

**Figure 7:**
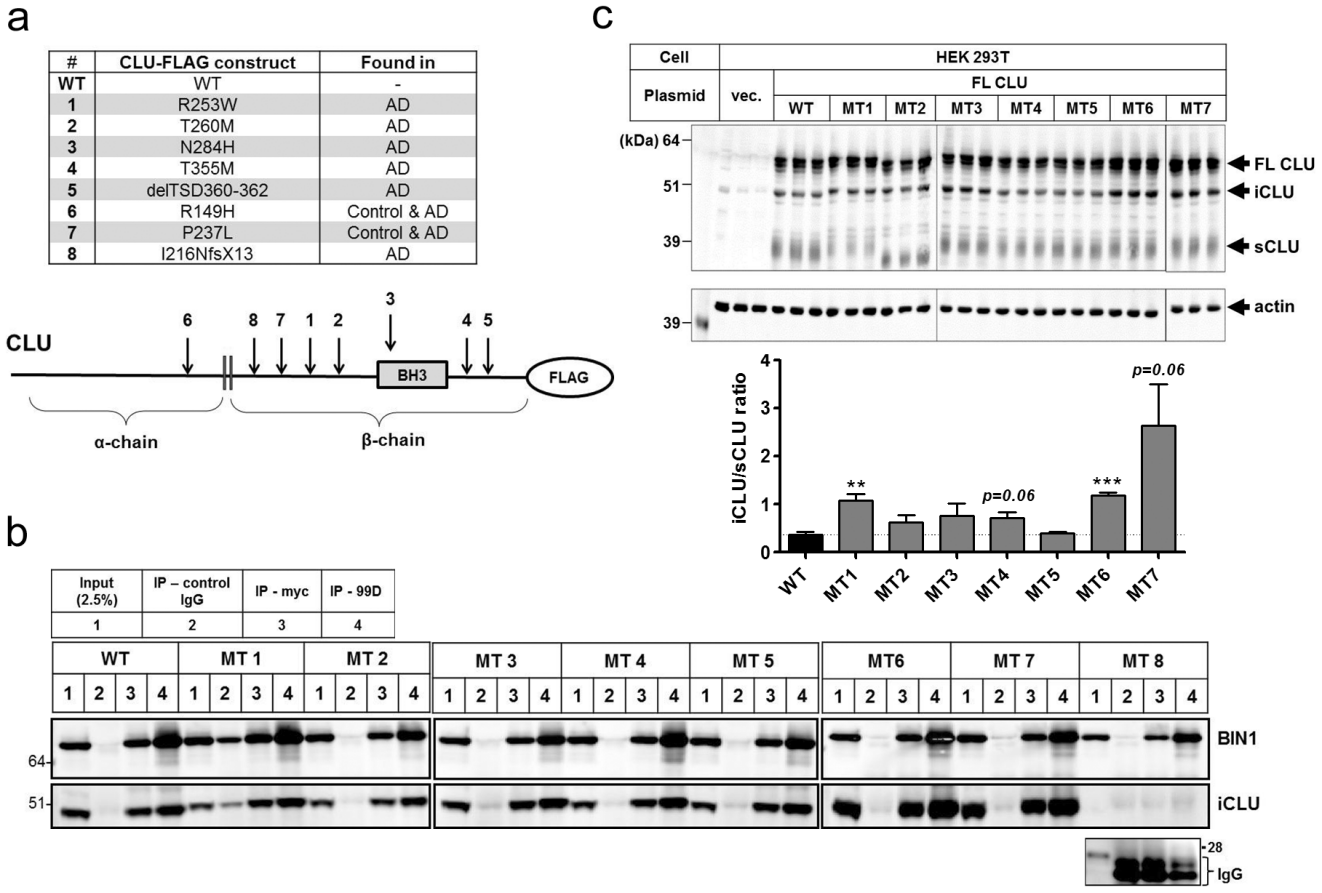
Analyses of coding *CLU* risk variants for interaction with BIN1.1 and CLU isoform expression. (a) Rare coding *CLU* variants recently linked to AD and construct design displaying mutations primarily localized within the β-chain of CLU. (b) Co-transfection experiments showed that while iCLU MT1–7 all appeared to interact with BIN1.1, the truncated iCLU MT8 lacking the *C*-terminus coiled-coil domain did not display binding to BIN1.1, indicating that iCLU and BIN1.1 association occurs via coiled-coil motifs. (c) FL-CLU mutants 1–7 were transfected in HEK 293T cells with subsequent analysis of iCLU and sCLU cellular levels by Western blotting. Semi-quantitative analysis of bands indicated that FL-CLU MT1 and MT6 generated increased levels of iCLU over sCLU when compared to WT CLU. **P<0.01; ***P<0.001, Mann-Whitney test.

Finally, we evaluated the interaction between BIN1.1 and eight rare CLU variants recently associated with AD [8]. The coding point mutations were introduced in the iCLU WT flag-tagged construct by site-direct mutagenesis (Fig. 7a). While CLU mutants 1–7 appear to interact equally with BIN1.1, a frame-shift CLU mutant lacking the *C*-terminus coiled-coil region failed to co-immunoprecipitate with BIN1.1 (Fig. 7b) further corroborating that iCLU and BIN1 association occurs via a coiled-coil interaction. Moreover, overexpression of full-length CLU mutants 1–7 in HEK 293T cells showed that certain coding mutations (MT1 and MT6) were associated with increased generation of iCLU relative to sCLU (Fig. 7c), suggesting a potential mechanism for pathogenicity of these variants.

## Discussion

After *APOE*, the *CLU* and *BIN1* genes have been identified as the most important susceptibility loci in late-onset AD [1]–[7], [33]–[37]. It has been hypothesized that CLU is involved in amyloid clearance, playing a protective role in AD. However, contrary to this premise, plasma levels of CLU correlate positively with AD severity and progression [50], and increased CLU mRNA expression is associated with a more deteriorated disease status [51]. It has been recently reported that elevated CSF clusterin contributes to entorhinal atrophy in patients with mild cognitive impairment and increased Aβ42 deposition [52], suggesting that clusterin might be involved in AD pathogenesis. In the present study we found a link between the *CLU rs11136000* SNP and CSF Tau levels in AD patients and provided evidence that intracellular forms of clusterin might play an important role in AD pathology. While the secreted form of CLU (sCLU) is induced during stress and inflammation and believed to be protective, intracellular forms of CLU previously linked to cytotoxicity are also upregulated under these conditions [12], [14]. Mechanisms induced by cellular stress, such as abnormal translocation of CLU from the ER/Golgi to the cytosol [12] and impaired degradation by the proteosome [14], as well as increased Aβ levels [17] and scrapie infection [53], all appear to drive accumulation of iCLU. Therefore *CLU* mutations that alter the ratio between iCLU and sCLU produced during stress could potentially modify risk for AD. In support of this hypothesis, we showed that certain AD-risk mutations in *CLU* significantly increased the ratio iCLU/sCLU following expression of the full-length CLU construct *in vitro*.

We reported here that overexpression of human Tau in the mouse brain was associated with a marked increase in iCLU levels before any pathological forms of Tau were detected, suggesting that iCLU and Tau are predicted to interact *in vivo*. However, because Tg4510 mice overexpress the frontotemporal dementia mutant P301L Tau, which has reduced ability to promote microtubule assembly and is more prone to aggregation than WT Tau [54] found in normal and AD patients, we performed reciprocal co-immunoprecipitation experiments comparing the interaction between iCLU and WT or P301L Tau in HEK 293T cells. Our results indicated that both Tau forms co-immunoprecipitated with iCLU when co-expressed in a cellular system and validated our conclusion that increased iCLU expression in Tg4510 mouse brain is indeed suggestive of a physiologically relevant interaction between iCLU and Tau.

Our cellular data supporting CLU as a novel Tau-interacting protein is similar to recent findings reported for BIN1 [39]. While iCLU interacted with both WT and P301L mutant full-length Tau, it did not co-immunoprecipitate the 4-repeat MBD of Tau alone, suggesting that the interaction between iCLU and Tau occurs outside the microtubule binding region. In addition, we showed that CLU and BIN1 co-immunoprecipitated primarily with Tau species found in neurofibrillary tangles from AD brains. Because initial findings from Chapuis *et al.* [39] suggest that BIN1 expression is detrimental, and increased expression of both CLU and brain-specific isoforms of BIN1 has been associated with AD status [51], we speculate that this newly discovered interaction between CLU and BIN1 could be related to AD risk via modulation of Tau function. CLU was previously shown to also interact via its coil-coiled motif with SCLIP, a member of the stathmin family proteins that modulate microtubule dynamics through association with tubulin [49]. Therefore, BIN1 expression levels could potentially modulate the interaction between iCLU and proteins regulating microtubule dynamics, including Tau.

A new interaction between CLU and coiled-coil-containing isoforms of neuronal BIN1 demonstrated here uncovers other putative mechanisms by which CLU and BIN1 could affect AD risk. It has been recently reported that the coiled-coil motif of neuronal BIN1 isoforms is also required for binding to dynamin 2, and that this interaction is essential for endocytosis [55]. Therefore, we hypothesize that increased intracellular CLU could also impair endocytosis by interfering with the interaction between BIN1 and dynamin 2. Endocytosis appears to be crucial in the etiology of AD as SNPs in genes encoding endocytosis-related proteins have been linked to the disease, including the clathrin adaptor *PICALM* [1]–[6] and *CD2AP* [36]. Interestingly, both PICALM and CD2AP have also been recently linked to Tau pathology [56], [57]. PICALM was found to co-localize and immunoprecipitate with hyperphosphorylated and misfolded Tau from brains of AD patients [53], while CD2AP was found to modulate Tau toxicity in *Drosophila* [57]. Knockdown of *cindr*, the fly ortholog of the human *CD2AP*, enhanced Tau-induced retinal toxicity [57], similarly to results observed for the fly ortholog of *BIN1* [39].

In summary, our novel findings provide groundwork for future studies that could shed light on the mechanisms by which GWAS-identified genes *CLU* and *BIN1* are linked to Tau pathology in AD, and help uncover new areas for therapeutic intervention.

## Materials and Methods

### Reagents and Antibodies

Reagents were purchased from Sigma-Aldrich unless specified otherwise. Antibodies used in this study were as follow: anti-APP 6E10 (SIG-39320, Covance), anti-Aβ42 12F4 (SIG-39142, Covance), mouse anti-Tau Tau12 (SIG-39416, Covance), mouse anti-Tau HT7 (MN1000, Thermo Scientific Pierce), mouse anti-phosphorylated Tau AT8 (MN1020, Thermo Scientific Pierce), rabbit anti-Tau H150 (SC-5587, Santa Cruz Biotechnology), rabbit anti-Tau (A0024, Dako), rabbit anti-clusterin H330 (SC-8354, Santa Cruz Biotechnology), goat anti-clusterin M18 (SC-6420, Santa Cruz Biotechnology), goat anti-clusterin C18 (SC-6419, Santa Cruz Biotechnology), mouse anti-BIN1 99D (05-449, Millipore), rabbit anti-Gapdh (G9545), rabbit anti-actin (A2066), and mouse anti-Flag (F1804), mouse anti-myc 9E10 (SC-40, Santa Cruz Biotechnology), mouse and goat IgG control (SC-2015 and SC-2028, Santa Cruz Biotechnology), donkey anti-mouse IgG (H+L) IRDye 800 (926-32212, LI-COR Biosciences), donkey anti-rabbit IgG (H+L) IRDye 680 (926-68073, LI-COR Biosciences), donkey anti-rabbit IgG (H+L) IRDye 800 (926-32213, LI-COR Biosciences), and donkey anti-goat IgG (H+L) IRDye 680 (926-68074, LI-COR Biosciences).

### Human Samples and Genotyping

All human samples were obtained from commercial sources and analyzed anonymously. Human blood and cerebrospinal fluid (CSF) from clinically diagnosed Alzheimer's disease patients and aged controls were purchased from PrecisionMed, Inc (www.precisionmed.com). CSF samples were aliquoted and stored at −80°C until analysis. Genomic DNA was extracted from blood cells using the QIAamp DNA blood midi prep (Qiagen). DNA samples were stored at −20°C until used. *APOE ε2/ε3/ε4* and *CLU rs11136000* polymorphisms were determined using polymerase chain reaction and restriction fragment length polymorphism (PCR-RFLP) method. All primers used for PCR were purchased from Applied Biosystems. PCR reagents included 2.5 µl of 500 nM of each primer, 25 ng of template genomic DNA, and 25 µl of AmpliTaq Gold 360 Master Mix (Applied Biosystems). For *APOE* genotyping, forward and reverse primer sequences were: 5′ ACAGAATTCGCCCCGGCCTGGTACAC 3′ and 5′ -TAAGCITGGCACGGCTGTCCAAGGA 3′. PCR products were digested with HhaI (New England Biolab) at 37°C for 1 hr, and the digested products were run in 20% TBE polyacrylamide gels. For genotyping the *CLU rs11136000* polymorphism, forward and reverse primer sequences were: 5′ CTTTGTAATGATGTACCATCTACCC 3′ and 5′ AGGCTGCAGACTCCCTGAAT 3′. PCR products were digested with ApoI (New England Biolab) at 37°C for 1 hr, and the digested products were run in 8% TBE polyacrylamide gels. Human brain tissue was obtained from National Disease Research Interchange (NDRI) and stored at −80°C until analysis.

### Quantification of CSF Aβ42, Tau and Clusterin

CSF Aβ42 was measured by a sandwich ELISA system using 6E10 as a capture antibody and alkaline phosphatase (AP)-conjugated 12F4 as a detection antibody. A 96-well black plate (Corning) was coated with the 6E10 monoclonal antibody at the concentration of 2 µg/ml in 0.05 M carbonate-bicarbonate buffer, pH 9.6, incubated overnight at 4°C with shaking, washed with PBS containing 0.05% Tween 20 and blocked with PBS containing 3% bovine serum albumin (BSA) for at least 24 hr at 4°C with mixing. CSF was diluted at 1∶4 with PBS containing 3% BSA. Diluted samples and a standard series of synthetic Aβ42 peptide (American Peptide Company) were incubated in triplicates with AP-conjugated detection antibody at 4°C overnight with shaking. After incubation, plates were washed and incubated with CDP-Star chemiluminescent substrate (Applied Biosystems) at RT for 20 min. The chemiluminescence was measured with an EnVision plate reader (Perkin Elmer). CSF total Tau was measured with INNOTEST hTAU Ag (Innogenetics) according to manufacturers' instructions. CSF clusterin (1∶200 dilution) was measured using a human clusterin ELISA kit (DCLU00, R&D Systems) according to manufactures' instructions.

### Animals

Tg4510 human Tau and Tg2576 human APP transgenic mouse lines were maintained at Taconic. Brains harvested from animals immediately after euthanasia with carbon dioxide were flash-frozen and stored at −80°C for subsequent analysis. All animals were handled according to the Public Health Service Policy on Humane Care and Use of Laboratory Animals guidelines and the study protocol was approved by the Institutional Animal Care and Use Committee of Merck Research Laboratories (Permit Number: 12089284580220).

### Plasmid cDNA

The pCMV6-XL5 empty vector was obtained from Origene. Full-length and intracellular human clusterin (FL CLU and iCLU, respectively) constructs were generated from human clusterin variant 1 cDNA clone (SC118977) purchased from Origene. Briefly, the first 52 and 85 amino acids of human clusterin isoform 1 (MQVC…RIGG and MQVC…ELQE, respectively) were deleted to generate FL CLU and iCLU, respectively, and the FLAG tag sequence (DYKDDDDK) was added at the *C*-terminus. Human BIN1 cDNA clones in a pCMV6-entry vector with myc-tag sequence (TRTRPLEQKLISEEDLAANDILDYKDDDDKV) at the *C*-terminus were obtained from Origene. Tau constructs (4R2N WT, 4R2N P301L, K18 WT and K18 P301L) were generated in house by subcloning into pcDNA4/TO vector from Life Technologies.

### Cell Culture and Transfection

Parental HEK 293T cells were obtained from the American Type Culture Collection (ATCC) and maintained in Dulbecco's Modified Eagle Medium (DMEM) containing 10% heat-inactivated fetal bovine serum (FBS) (Life Technologies). HEK 293T cells stably expressing inducible human Tau 4R2N (iTau-HEK) were developed at Origene. iTau-HEK cells were maintained in DMEM containing 10% Tet system approved FBS (Clontech), 1× non-essential amino acids (Life Technologies), 200 µg/mL hygromycin B (Life Technologies) and 400 µg/mL geneticin (Life Technologies) at 37°C with 5% CO_2_. Tau expression was induced by incubating iTau-HEK cells in growth media containing 1 µg/mL doxycycline (Clontech) for 48–72 hr. Transient transfections of CLU and Tau constructs were performed using the Lipofectamine LTX reagent (Life Technologies) according to the manufacturers' instructions. Cells were harvested 48–72 hr after transfection for subsequent analyses.

### Subcellular fractionation

Subcellular fractionation of cells and brain tissue was performed using the Qproteome Cell Compartment Kit (Qiagen) according to the manufacturers' instructions. Briefly, cell pellets (∼5×10^6^ cells) or brain tissue (20–30 mg) were solubilized in lysis buffer and centrifuged at 1,000 g for 10 min at 4°C. Cytosolic proteins were obtained from the supernatant. The remaining pellet was resuspended in ice-cold extraction buffer CE2 and centrifuged at 6,000 g for 10 min at 4°C. Membrane proteins were obtained from the supernatant. Subsequent pellet was resuspended in benzonase nuclease and extraction buffer CE3 and centrifuged at 6,800 g for 10 min at 4°C. Nuclear proteins were obtained from the supernatant. The final pellet was resuspended in extraction buffer CE4 to obtain cytoskeletal/insoluble proteins.

### Western blotting and Immunoprecipitation

Tissue samples were homogenized in NP-40 lysis buffer (10 mM HEPES, pH 7.4; 140 mM NaCl; 0.5% NP40) or in Triton lysis buffer containing 1% Triton X-100 in phosphate buffered saline (PBS) supplemented with Complete protease inhibitor cocktail (Roche Applied Sciences) and PhosSTOP phosphatase inhibitor cocktail (Roche Applied Sciences) using a TissueLyzer. Cells were washed briefly with ice-cold PBS and lysed in NP-40 or Triton lysis buffer supplemented with Complete protease inhibitor and PhosSTOP phosphatase inhibitor cocktails. All lysates were solubilized at 4°C for 1–3 hr and cleared by centrifugation at 10,000 g for 10 min. Protein concentration was determined by the BCA protein assay kit (Pierce). For Western blotting analysis, lysates were heated at 70°C for 10 min in NuPAGE LDS sample buffer and NuPAGE sample reducing agent (Life Technologies), and resolved on NuPAGE Novex Bis-Tris gel (Life Technologies). Membranes were immunoblotted with appropriate antibodies and analyzed with the Odyssey infrared imaging system (LI-COR). For immunoprecipitation, protein G-coated dynabeads (Life Technologies) were coated with appropriate antibodies at a concentration of 5 µg of antibody per 50 µl of dynabeads. Tissue or cell lysates were incubated with antibody-coated dynabeads for 30 min at RT. After immunoprecipitation, samples were suspended in 1× NuPAGE LDS sample buffer and NuPAGE sample reducing agent and analyzed by Western blotting.

### Primary neuronal and astrocyte cultures

Human neurons (Cat# 1520) and astrocytes (Cat #1801) were obtained from ScienCell Research. Briefly, neurons were plated in 6-well poly-L-lysine-coated culture plates (Thermo 152035) according to manufactures' protocol. After 14 days in culture, neurons were washed briefly with ice-cold PBS and lysed in RIPA buffer (Thermo 89900) supplemented with Complete protease inhibitor and PhosSTOP phosphatase inhibitor cocktails (Roche Applied Sciences). For human astrocytes, cells were cultured following manufacture instructions and 0.5×10^6^ cells were sub-cultured in 6-well poly-L-lysine-coated culture plates for 24 hrs. Astrocytes were then washed and lysed in the same manner as human neurons. Lysates were solubilized at 4°C for 1 hr and cleared by centrifugation at 16,000 g for 5 min. Protein concentration was determined by the BCA protein assay kit (Pierce).

### Statistical analysis

Receiver operating characteristic curve (ROC) analysis was performed using JMP 11 (SAS Institute). Group differences for each analyte were assessed by the Student's *t* test for two-group comparisons and the One-way ANOVA test for multiple comparisons using GraphPad Prism 5.0 (GraphPad Software Inc.). For comparison of categorical parameters, the Pearson's chi-square test was used. A *P* value of less than or equal to 0.05 was considered statistically significant.

## References

[1] Harold D, Abraham R, Hollingworth P, Sims R, Gerrish A, et al (2009) Genome-wide association study identifies variants at CLU and PICALM associated with Alzheimer's disease. Nat Genet 41: 1088–1093. 19734902 10.1038/ng.440 PMC2845877

[2] Lambert JC, Heath S, Even G, Campion D, Sleegers K, et al (2009) Genome-wide association study identifies variants at CLU and CR1 associated with Alzheimer's disease. Nat Genet 41: 1094–1099. 19734903 10.1038/ng.439

[3] Carrasquillo MM, Belbin O, Hunter TA, Ma L, Bisceglio GD, et al (2010) Replication of CLU, CR1, and PICALM associations with Alzheimer's disease. Arch Neurol 67: 961–964. 20554627 10.1001/archneurol.2010.147 PMC2919638

[4] Corneveaux JJ, Myers AJ, Allen AN, Pruzin JJ, Ramirez M, et al (2010) Association of CR1, CLU and PICALM with Alzheimer's disease in a cohort of clinically characterized and neuropathologically verified individuals. Hum Mol Genet 19: 3295–3301. 20534741 10.1093/hmg/ddq221 PMC2908469

[5] Jun G, Naj AC, Beecham GW, Wang LS, Buros J, et al (2010) Meta-analysis confirms CR1, CLU, and PICALM as alzheimer disease risk loci and reveals interactions with APOE genotypes. Arch Neurol 67: 1473–1484. 20697030 10.1001/archneurol.2010.201 PMC3048805

[6] Lee JH, Cheng R, Barral S, Reitz C, Medrano M, et al (2011) Identification of novel loci for Alzheimer disease and replication of CLU, PICALM, and BIN1 in Caribbean Hispanic individuals. Arch Neurol 68: 320–328. 21059989 10.1001/archneurol.2010.292 PMC3268783

[7] Wijsman EM, Pankratz ND, Choi Y, Rothstein JH, Faber KM, et al (2011) Genome-wide association of familial late-onset Alzheimer's disease replicates BIN1 and CLU and nominates CUGBP2 in interaction with APOE. PLoS Genet 7: e1001308. 21379329 10.1371/journal.pgen.1001308 PMC3040659

[8] Bettens K, Brouwers N, Engelborghs S, Lambert JC, Rogaeva E, et al (2012) Both common variations and rare non-synonymous substitutions and small insertion/deletions in CLU are associated with increased Alzheimer risk. Mol Neurodegener 7: 3–14. 22248099 10.1186/1750-1326-7-3 PMC3296573

[9] Nuutinen T, Suuronen T, Kauppinen A, Salminen A (2009) Clusterin: a forgotten player in Alzheimer's disease. Brain Res Rev 61: 89–104. 19651157 10.1016/j.brainresrev.2009.05.007

[10] de Silva HV, Harmony JA, Stuart WD, Gil CM, Robbins J (1990) Apolipoprotein J: structure and tissue distribution. Biochemistry 5: 5380–5389. 10.1021/bi00474a025 1974459

[11] Calero M, Tokuda T, Rostagno A, Kumar A, Zlokovic B, et al (1999) Functional and structural properties of lipid-associated apolipoprotein J (clusterin). Biochem J 344: 375–383. 10567218 PMC1220653

[12] Nizard P, Tetley S, Le Dréan Y, Watrin T, Le Goff P, et al (2007) Stress-induced retrotranslocation of clusterin/ApoJ into the cytosol. Traffic 8: 554–565. 17451556 10.1111/j.1600-0854.2007.00549.x

[13] Bettuzzi S, Rizzi F (2009) Nuclear CLU (nCLU) and the fate of the cell. Adv Cancer Res 104: 59–88. 19878773 10.1016/S0065-230X(09)04005-6

[14] Prochnow H, Gollan R, Rohne P, Hassemer M, Koch-Brandt C, et al (2013) Non-secreted clusterin isoforms are translated in rare amounts from distinct human mRNA variants and do not affect Bax-mediated apoptosis or the NF-κB signaling pathway. PLoS One 8: e75303. 24073260 10.1371/journal.pone.0075303 PMC3779157

[15] Zhang H, Kim JK, Edwards CA, Xu Z, Taichman R, et al (2005) Clusterin inhibits apoptosis by interacting with activated Bax. Nat Cell Biol 7: 909–915. 16113678 10.1038/ncb1291

[16] Kim N, Yoo JC, Han JY, Hwang EM, Kim YS, et al (2011) Human nuclear clusterin mediates apoptosis by interacting with Bcl-XL through C-terminal coiled coil domain. J Cell Physiol 227: 1157–1167. 10.1002/jcp.22836 21567405

[17] Killick R, Ribe EM, Al-Shawi R, Malik B, Hooper C, et al (2014) Clusterin regulates β-amyloid toxicity via Dickkopf-1-driven induction of the wnt-PCP-JNK pathway. Mol Psychiatry 19: 88–98. 23164821 10.1038/mp.2012.163 PMC3873038

[18] Duguid JR, Bohmont CW, Liu NG, Tourtellotte WW (1989) Changes in brain gene expression shared by scrapie and Alzheimer disease. Proc Natl Acad Sci USA 86: 7260–7264. 2780570 10.1073/pnas.86.18.7260 PMC298037

[19] May PC, Lampert-Etchells M, Johnson SA, Poirier J, Masters JN (1990) Dynamics of gene expression for a hippocampal glycoprotein elevated in Alzheimer's disease and in response to experimental lesions in rat. Neuron 5: 831–839. 1702645 10.1016/0896-6273(90)90342-d

[20] McGeer PL, Kawamata T, Walker DG (1992) Distribution of clusterin in Alzheimer brain tissue. Brain Res 579: 337–341. 1378350 10.1016/0006-8993(92)90071-g

[21] Lidström AM, Bogdanovic N, Hesse C, Volkman I, Davidsson P, et al (1998) Clusterin (apolipoprotein J) protein levels are increased in hippocampus and in frontal cortex in Alzheimer's disease. Exp Neurol 154: 511–521. 9878186 10.1006/exnr.1998.6892

[22] DeMattos RB, O'dell MA, Parsadanian M, Taylor JW, Harmony JA, et al (2002) Clusterin promotes amyloid plaque formation and is critical for neuritic toxicity in a mouse model of Alzheimer's disease. Proc Natl Acad Sci USA 99: 10843–10848. 12145324 10.1073/pnas.162228299 PMC125060

[23] DeMattos RB, Cirrito JR, Parsadanian M, May PC, O'Dell MA, et al (2004) ApoE and clusterin cooperatively suppress Abeta levels and deposition: evidence that ApoE regulates extracellular Abeta metabolism in vivo. Neuron 41: 193–202. 14741101 10.1016/s0896-6273(03)00850-x

[24] Castellano JM, Kim J, Stewart FR, Jiang H, DeMattos RB, et al (2011) Human apoE isoforms differentially regulate brain amyloid-β peptide clearance. Sci Transl Med 3: 89ra57. 10.1126/scitranslmed.3002156 PMC3192364 21715678

[25] Narayan P, Meehan S, Carver JA, Wilson MR, Dobson CM, et al (2012) Amyloid-β oligomers are sequestered by both intracellular and extracellular chaperones. Biochemistry 51: 9270–9276. 23106396 10.1021/bi301277k PMC4981287

[26] Cascella R, Conti S, Tatini F, Evangelisti E, Scartabelli T, et al (2013) Extracellular chaperones prevent Aβ42-induced toxicity in rat brains. Biochim Biophys Acta 1832: 1217–1226. 23602994 10.1016/j.bbadis.2013.04.012

[27] Baig S, Palmer LE, Owen MJ, Williams J, Kehoe PG, et al (2012) Clusterin mRNA and protein in Alzheimer's disease. J Alzheimers Dis 28: 337–344. 22232000 10.3233/JAD-2011-110473

[28] Hughes TM, Lopez OL, Evans RW, Kamboh MI, Williamson JD, et al (2014) Markers of cholesterol transport are associated with amyloid deposition in the brain. Neurobiol Aging 35: 802–807. 24199960 10.1016/j.neurobiolaging.2013.09.040 PMC3896052

[29] Braskie MN, Jahanshad N, Stein JL, Barysheva M, McMahon KL, et al (2011) Common Alzheimer's disease risk variant within the CLU gene affects white matter microstructure in young adults. J Neurosci 31: 6764–6770. 21543606 10.1523/JNEUROSCI.5794-10.2011 PMC3176803

[30] Erk S, Meyer-Lindenberg A, Opitz von Boberfeld C, Esslinger C, Schnell K, et al (2011) Hippocampal function in healthy carriers of the CLU Alzheimer's disease risk variant. J Neurosci 31: 18180–18184. 22159129 10.1523/JNEUROSCI.4960-11.2011 PMC6634131

[31] Thambisetty M, Beason-Held LL, An Y, Kraut M, Nalls M, et al (2013) Alzheimer risk variant CLU and brain function during aging. Biol Psychiatry 73: 399–405. 22795969 10.1016/j.biopsych.2012.05.026 PMC3488132

[32] Sakamuro D, Elliott KJ, Wechsler-Reya R, Prendergast GC (1996) BIN1 is a novel MYC-interacting protein with features of a tumour suppressor. Nat Genet 14: 69–77. 8782822 10.1038/ng0996-69

[33] Seshadri S, Fitzpatrick AL, Ikram MA, DeStefano AL, Gudnason V, et al (2010) Genome-wide analysis of genetic loci associated with Alzheimer disease. JAMA 303: 1832–1840. 20460622 10.1001/jama.2010.574 PMC2989531

[34] Carrasquillo MM, Belbin O, Hunter TA, Ma L, Bisceglio GD, et al (2011) Replication of BIN1 association with Alzheimer's disease and evaluation of genetic interactions. J Alzheimers Dis 24: 751–758. 21321396 10.3233/JAD-2011-101932 PMC3489170

[35] Hu X, Pickering E, Liu YC, Hall S, Fournier H, et al (2011) Meta-analysis for genome-wide association study identifies multiple variants at the BIN1 locus associated with late-onset Alzheimer's disease. PLoS One 6: e16616. 21390209 10.1371/journal.pone.0016616 PMC3044719

[36] Naj AC, Jun G, Beecham GW, Wang LS, Vardarajan BN, et al (2011) Common variants at MS4A4/MS4A6E, CD2AP, CD33 and EPHA1 are associated with late-onset Alzheimer's disease. Nat Genet 43: 436–441. 21460841 10.1038/ng.801 PMC3090745

[37] Logue MW, Schu M, Vardarajan BN, Buros J, Green RC, et al (2011) A comprehensive genetic association study of Alzheimer disease in African Americans. Arch Neurol 68: 1569–1579. 22159054 10.1001/archneurol.2011.646 PMC3356921

[38] Tan MS, Yu JT, Tan L (2013) Bridging integrator 1 (BIN1): form, function, and Alzheimer's disease. Trends Mol Med 19: 594–603. 23871436 10.1016/j.molmed.2013.06.004

[39] Chapuis J, Hansmannel F, Gistelinck M, Mounier A, Van Cauwenberghe C, et al (2013) Increased expression of BIN1 mediates Alzheimer genetic risk by modulating Tau pathology. Mol Psychiatry 18: 1225–1234. 23399914 10.1038/mp.2013.1 PMC3807661

[40] Cruchaga C, Ebbert MT, Kauwe JS (2014) Genetic discoveries in AD using CSF amyloid and tau. Curr Genet Med Rep 2: 23–29. 24729949 10.1007/s40142-014-0031-0 PMC3979575

[41] Fagan AM, Shaw LM, Xiong C, Vanderstichele H, Mintun MA, et al (2011) Comparison of analytical platforms for cerebrospinal fluid measures of β-amyloid 1–42, total tau, and p-tau181 for identifying Alzheimer disease amyloid plaque pathology. Arch Neurol 68: 1137–1144. 21555603 10.1001/archneurol.2011.105 PMC3154969

[42] Schoonenboom NS, Reesink FE, Verwey NA, Kester MI, Teunissen CE, et al (2012) Cerebrospinal fluid markers for differential dementia diagnosis in a large memory clinic cohort. Neurology 78: 47–54. 22170879 10.1212/WNL.0b013e31823ed0f0

[43] Schjeide BM, Schnack C, Lambert JC, Lill CM, Kirchheiner J, et al (2011) The role of clusterin, complement receptor 1, and phosphatidylinositol binding clathrin assembly protein in Alzheimer disease risk and cerebrospinal fluid biomarker levels. Arch Gen Psychiatry 68: 207–213. 21300948 10.1001/archgenpsychiatry.2010.196

[44] Elias-Sonnenschein LS, Bertram L, Visser PJ (2012) Relationship between genetic risk factors and markers for Alzheimer's disease pathology. Biomark Med 6: 477–495. 22917148 10.2217/bmm.12.56

[45] Nilselid AM, Davidsson P, Nägga K, Andreasen N, Fredman P, et al (2006) Clusterin in cerebrospinal fluid: analysis of carbohydrates and quantification of native and glycosylated forms. Neurochem Int 48: 718–728. 16490286 10.1016/j.neuint.2005.12.005

[46] Sihlbom C, Davidsson P, Sjögren M, Wahlund LO, Nilsson CL (2008) Structural and quantitative comparison of cerebrospinal fluid glycoproteins in Alzheimer's disease patients and healthy individuals. Neurochem Res 33: 1332–1340. 18288611 10.1007/s11064-008-9588-x

[47] Ramsden M, Kotilinek L, Forster C, Paulson J, McGowan E, et al (2005) Age-dependent neurofibrillary tangle formation, neuron loss, and memory impairment in a mouse model of human tauopathy (P301L). J Neurosci 25: 10637–10647. 16291936 10.1523/JNEUROSCI.3279-05.2005 PMC6725849

[48] Yang CR, Leskov K, Hosley-Eberlein K, Criswell T, Pink JJ, et al (2000) Nuclear clusterin/XIP8, an x-ray-induced Ku70-binding protein that signals cell death. Proc Natl Acad Sci USA 97: 5907–5912. 10823943 10.1073/pnas.97.11.5907 PMC18532

[49] Kang SW, Shin YJ, Shim YJ, Jeong SY, Park IS, et al (2005) Clusterin interacts with SCLIP (SCG10-like protein) and promotes neurite outgrowth of PC12 cells. Exp Cell Res 309: 305–315. 16038898 10.1016/j.yexcr.2005.06.012

[50] Schrijvers EM, Koudstaal PJ, Hofman A, Breteler MM (2011) Plasma clusterin and the risk of Alzheimer disease. JAMA 305: 1322–1326. 21467285 10.1001/jama.2011.381

[51] Karch CM, Jeng AT, Nowotny P, Cady J, Cruchaga C, et al (2012) Expression of novel Alzheimer's disease risk genes in control and Alzheimer's disease brains. PLoS One 7: e50976. 23226438 10.1371/journal.pone.0050976 PMC3511432

[52] Desikan RS, Thompson WK, Holland D, Hess CP, Brewer JB, et al (2014) The Role of Clusterin in Amyloid-β-Associated Neurodegeneration. JAMA Neurol 71: 180–187. 24378367 10.1001/jamaneurol.2013.4560 PMC4118752

[53] Kang SW, Yoon SY, Park JY, Kim DH (2013) Unglycosylated clusterin variant accumulates in the endoplasmic reticulum and induces cytotoxicity. Int J Biochem Cell Biol 45: 221–231. 23201481 10.1016/j.biocel.2012.11.014

[54] Barghorn S, Zheng-Fischhöfer Q, Ackmann M, Biernat J, von Bergen M, et al (2000) Structure, microtubule interactions, and paired helical filament aggregation by tau mutants of frontotemporal dementias. Biochemistry 39: 11714–11721. 10995239 10.1021/bi000850r

[55] Ellis JD, Barrios-Rodiles M, Colak R, Irimia M, Kim T, et al (2012) Tissue-specific alternative splicing remodels protein-protein interaction networks. Mol Cell 46: 884–892. 22749401 10.1016/j.molcel.2012.05.037

[56] Ando K, Brion JP, Stygelbout V, Suain V, Authelet M, et al (2013) Clathrin adaptor CALM/PICALM is associated with neurofibrillary tangles and is cleaved in Alzheimer's brains. Acta Neuropathol 125: 861–878. 23589030 10.1007/s00401-013-1111-z

[57] Shulman JM, Imboywa S, Giagtzoglou N, Powers MP, Hu Y, et al (2014) Functional screening in Drosophila identifies Alzheimer's disease susceptibility genes and implicates Tau-mediated mechanisms. Hum Mol Genet 23: 870–877. 24067533 10.1093/hmg/ddt478 PMC3900103

